# Acknowledgement to Reviewers of *Biosensors* in 2013

**DOI:** 10.3390/bios4010045

**Published:** 2014-02-27

**Authors:** Biosensors Editorial Office

**Affiliations:** MDPI AG Klybeckstrasse 64 CH-4057 Basel Switzerland

The editors of *Biosensors* would like to express their sincere gratitude to the following reviewers for assessing manuscripts in 2013:

Antonelli, Marta Letizia
Aono, Shigetoshi
Bedioui, Fethi
Benoukraf, Touati
Berezin, Mikhail Y.
Bond, Tiziana
Le Brun, Nick E.
Cano, Amparo
Catellani, Marinella
Chen, Chien-ming
Cheng, Xuanhong
Chiu, Nan-Fu
D'arrigo, Cristina
De Coninck, Joël
Duan, Jicheng
Engel, Yoni
Erler, Janine T.
Flögel, Ulrich
Fossey, John S.
Gao, Zhiqiang
Georgakopoulos, Dimitrios G.
Giouroudi, Ioanna
Graham, E. Scott
Gutierrez Gallego, Ricardo
Hall, Drew A.
Hamon, Jacques
Haupt, Karsten
Hicks, Barry W.
Ho, Ho-Pui
Huang, Xiaohua
Huber, Walter
Huh, Yun Suk
Ikeda-Saito, Masao
Jiang, Nan
Judge, Kevin
Jung, Friedrich
Kobayashi, Kazuo
Koçer, Armagan
Kothe, Erika
Kung, Ching
Kustermann, Stefan
Lee, Kwan Hyi
Liao, Jiayu
Liu, Songqin
Lou, Xia
Mamotte, Cyril D.
Matsui, Jun
Meulenberg, Eline P.
Mitchell, John
Moon, Kee S.
Niles, Andrew L.
Nugen, Sam R.
Oh, Byung-Keun
Ortiz De Orué Lucana, Dario
Ozcan, Aydogan
Park, Hun-kuk
Park, Tae Jung
Petty, Jeffrey T.
Phambu, Nsoki
Popp, Jürgen
Poulos, Thomas L.
Pourdeyhimi, Behnam
Ramadan, Qasem
Rooney, Denise
Sagle, Laura
Salmain, Michèle
Sandros, Marinella G.
Schalk, Isabelle J.
Shamsi, Mohtashim Hassan
Singaram, Bakthan
Sousa, Eduardo H. S.
Srinivas, Mangala
Sun, Daochun
Suriyaranayaranan, Subramanian
Tabb, Joel S.
Tappura, Kirsi
Tsai, Wen-Chi
Vosch, Tom
Walmsley, Richard
Wang, Hsiang-Chen
Wang, Yuhong
Watts, Kylie J.
Weinert, Emily
Willner, Itamar
Wu, Hao
Xiong, Xiangling
Yang, Hong-chang
Zadran, Sohila
Zhang, Ze
Zuchner, Thole

